# Trends in Complementary Feeding Indicators in Children Aged 6–23 Months According to Participation in a Conditional Cash Transfer Program: Data from the Brazilian Food and Nutrition Surveillance System, 2015–2019

**DOI:** 10.3390/ijerph21070923

**Published:** 2024-07-15

**Authors:** Andreia Andrade-Silva, Dayana Rodrigues Farias, Thais Rangel Bousquet Carrilho, Inês Rugani Ribeiro de Castro, Gilberto Kac, Maria Beatriz Trindade de Castro

**Affiliations:** 1 Department of Social and Applied Nutrition, Josué de Castro Institute of Nutrition, Federal University of Rio de Janeiro, Rio de Janeiro 21941-902, Brazil; 2 Nutritional Epidemiology Observatory, Department of Social and Applied Nutrition, Josué de Castro Institute of Nutrition, Federal University of Rio de Janeiro, Rio de Janeiro 21941-902, Brazil; dayana.farias@nutricao.ufrj.br (D.R.F.);; 3 Department of Obstetrics & Gynaecology, Faculty of Medicine, University of British Columbia, Vancouver, BC V6H3N1, Canada; 4 Department of Social Nutrition, Institute of Nutrition, State University of Rio de Janeiro, Rio de Janeiro 20550-013, Brazil

**Keywords:** child, food intake, ultra-processed foods, public health surveillance

## Abstract

Inadequate practices during complementary feeding are associated with malnutrition, especially in children experiencing vulnerable conditions and social inequality. The aim of this study was to evaluate the trends in complementary feeding indicators (CFIs) according to participation in a Brazilian cash transferu program—the *Bolsa Família* Program (BFP). This was a time-series study with secondary data from 600,138 children assisted from 2015 to 2019 and registered within the Brazilian Food and Nutrition Surveillance System. The CFIs assessed were food introduction, minimum meal frequency and appropriate consistency, minimum dietary diversity, iron-rich food, vitamin A-rich food, ultra-processed food consumption, and zero vegetable or fruit consumption. Prevalence and 95% confidence intervals were calculated for the CFIs according to BFP, the region of residence, and the child’s age. The Prais–Winsten regression method was used to analyze the temporal trend. There was a steady trend for all CFIs of a healthy diet. A decrease in ultra-processed food consumption for both BFP (−10.02%) and non-BFP children (−9.34%) was observed over the years. Children residing in the North and Northeast regions and those enrolled in the BFP were more distant from the recommended feeding practices when compared to the other regions and non-BFP children. The results highlight the relevance of nutritional surveillance and the need to improve food and nutrition public policies for children aged 6–23 months, particularly for those experiencing greater social vulnerability.

## 1. Introduction

There is evidence of a triple burden of malnutrition in children under five years of age—undernutrition, overnutrition, and micronutrient deficiencies—as a consequence of poor-quality feeding. This triple burden may lead to stunting, reduced cognitive skills, low immunity, and a higher risk of infections and death, as well as long-term conditions such as obesity, diabetes, and metabolic disorders [1]. Among children under 24 months, inadequate practices during complementary feeding, such as the early introduction of sugar and ultra-processed foods such as sweets, candies, sugar-sweetened beverages, instant noodles, sandwich cookies, and savory snacks [2,3] and low consumption of fruits and vegetables [4], are associated with malnutrition. According to the Brazilian National Survey on Child Nutrition (ENANI-2019), 80.5% of children aged 6–23 months consumed ultra-processed foods and only 63.4% achieved the minimum dietary diversity on the day before the interview [5].

The inadequacy of complementary feeding seems to be determined by some sociodemographic characteristics, especially those related to social vulnerability [6,7,8,9,10]. The *Bolsa Família* Program (BFP) is a conditional cash transfer program created in 2003 for families living in poverty, extreme poverty, conditions of vulnerability, and social inequality [11]. BFP children are monitored by the Brazilian Food and Nutrition Surveillance System (SISVAN), which is a national administrative system implemented since 1990. Information on anthropometric variables, food consumption markers, and sociodemographic characteristics routinely collected in primary health care are registered in SISVAN [12]. Some local studies have shown the early introduction of ultra-processed foods in children benefiting from the BFP [13,14].

The World Health Organization (WHO) and The Brazilian Ministry of Health recommends exclusive breastfeeding during the first six months of life, and continued breastfeeding until at least 24 months [15,16]. Appropriate and timely complementary feeding practices should begin around six months of age and should provide healthy, safe, and diversified foods to ensure enough energy and nutrients for adequate growth and development during the first two years [17,18]. The use of complementary feeding indicators is recommended to monitor the diet of children aged 6–23 months [19].

However, there is a lack of information on complementary feeding indicators of Brazilian children under two years of age, who are beneficiaries of the BFP, and how they are distributed over time to help predict trends. Therefore, this study aimed to evaluate the trends in complementary feeding indicators in Brazilian children aged 6–23 months according to participation in the BFP and sociodemographic characteristics from 2015 to 2019.

## 2. Materials and Methods

### 2.1. Design and Study Population

This is a time-series study with microdata from the Brazilian Food and Nutrition Surveillance System (SISVAN) comprising children aged 6–23 months from all geographic regions of Brazil from 2015 to 2019. In SISVAN, data are collected during the primary health care routine by health care professionals following standardized protocols and are registered in the system by authorized professionals [12]. The Ministry of Health recommends that anthropometric and food intake data be collected at 6, 9, 12, 18, and 24 months for children aged 6–24 months [12].

In 2015, the food intake assessment method was updated, which does not allow comparisons with the data collected in the previous period. Additionally, a recent study revealed that the SISVAN coverage in 2020 was negatively impacted due to the COVID-19 pandemic restrictions [20].

### 2.2. Database Linkage and Data Cleaning Process

**Figure 1:**
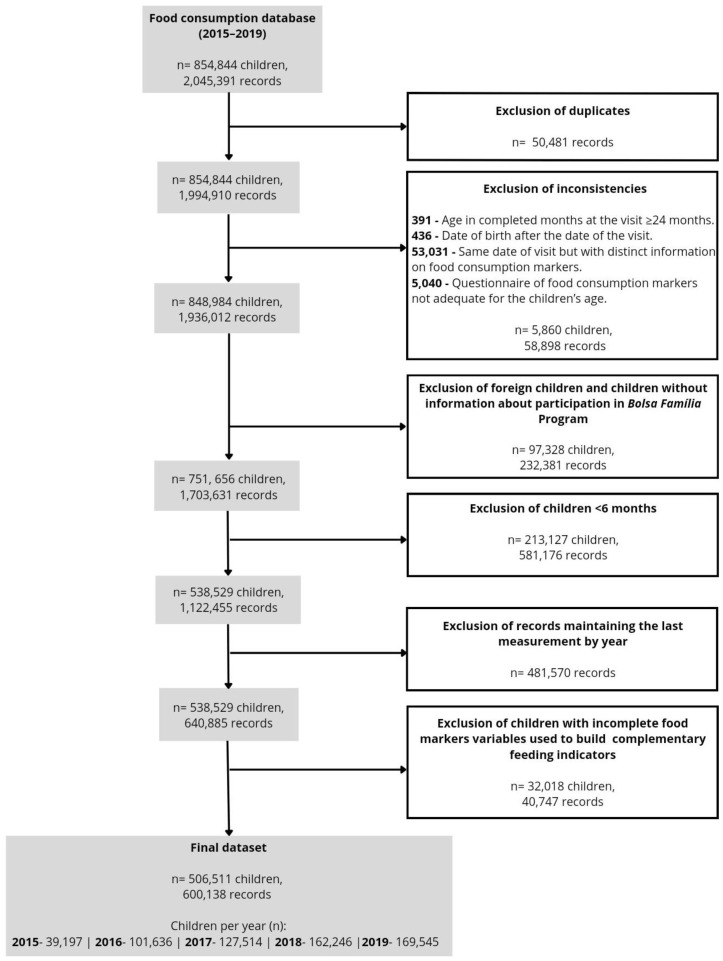
Flowchart for the constitution of the dataset used in the study.

The SISVAN food consumption database from 2015 to 2019 had 2,045,391 records from 854,844 children. Data cleaning procedures were performed with the aim of improving the quality of the data. The first step was the identification and exclusion of duplicate entries, resulting in the exclusion of 50,481 records. Then, the data consistency was checked. There were exclusions when (i) the age in completed months at the visit was ≥ 24 months; (ii) the date of birth was registered as having occurred after the date of the visit; (iii) there were duplicate lines for the exact date of visit but with specific information on food consumption markers (both lines were excluded); (iv) when the questionnaire related to food consumption markers was not adequate for the child’s age (Figure 1).

For the analysis, Brazilian children aged 6–23 months with data related to BFP participation and the construction of the complementary feeding indicators were considered eligible. Then, 1,703,631 records from 751,656 Brazilian children with information about the BFP remained, and children under 6 months were excluded (*n* = 213,127). If repeated measurements were taken from the same child within a given year, only the last measurement of each year was included, resulting in 640,885 observations from 538,529 children. Furthermore, children with missing data on the food markers used to build the indicators (*n* = 32,018 children, 40,747 records) were excluded. The final sample comprised 506,511 children and 600,138 records (Figure 1).

### 2.3. Complementary Feeding Indicators and Sociodemographic Variables

Complementary feeding was assessed according to the indicators proposed by the Brazilian Ministry of Health [19] as follows: food introduction at 6–8 months (FI), minimum meal frequency and appropriate consistency (MMF), minimum dietary diversity (MDD), iron-rich food consumption (IFC), vitamin A-rich food consumption (VAFC), and ultra-processed food consumption (UFC). The indicator zero vegetable or fruit consumption (ZVF) [21] was also included. Other indicators proposed by the World Health Organization (WHO) [21] were also calculated as follows: introduction of solid, semi-solid, or soft foods at 6–8 months (ISSSF), unhealthy food consumption (UNFC), and egg and/or flesh food consumption (EFF). The definition of each indicator is depicted in Table S1.

The data used to calculate the complementary feeding indicators were obtained from the SISVAN questionnaire, which identifies food consumption markers on the day before the dietary assessment. The food consumption markers in the questionnaire were as follows: breast milk, meal frequency and consistency, other types of milk (except breast milk), porridge with cow’s milk, yogurt, orange-colored vegetables/fruits or dark green leaves, other vegetables, other fruits, meat/eggs, liver, legumes, cereals/tubers, hamburgers/sausages, sugar-sweetened beverages, instant noodles/packaged snacks/crackers, and sandwich cookies/sweets/candies (their answer options as well as each food marker used to construct the indicators are shown in Supplementary Table S2). Answers of “don’t know” were considered missing data. We adopted the term “meals” to refer to solid, semi-solid, and soft foods offered as lunch and dinner [16].

The data were stratified according to the following sociodemographic variables: geographic region of residence in Brazil (North, Northeast, Southeast, South, and Midwest), children’s age groups (6–11, 12–17, and 18–23 months), and participation in the BFP (beneficiaries of the BFP [BBFP] or non-beneficiaries of the BFP [NBFP]).

### 2.4. Statistical Analysis

Prevalence and 95% confidence intervals (95% CI) were calculated for the sociodemographic variables according to participation in the BFP and for the indicators according to the BFP and sociodemographic variables in each year (2015, 2016, 2017, 2018, and 2019). Prevalence differences were considered statistically significant when there was no overlap of the 95% CI. The analyses were performed on an institutional server that remotely processes large volumes of data through the JupyterHub platform. All analyses were conducted using the STATA software, version 15 [22].

Time trend analysis was performed using Prais–Winsten regression, which is recommended for time series studies [23]. The prevalence of each indicator was converted into a logarithmic scale and was considered as the dependent variable, and the year was considered the independent variable. The β coefficient and respective 95% CI of the regression were included in the formula used to define the annual percentage variation (APC) = (−1 + 10^β^) × 100 and its 95% CI = (−1 + 10^βminimum^) × 100; (−1 + 10^βmaximum^) × 100. When APC was positive, the time series was considered an upward trend; when negative, it was a downward trend; and when there was no significant difference from zero, it was stationary [23].

## 3. Results

The study sample included 600,138 children: 39,197 in 2015; 101,636 in 2016; 127,514 in 2017; 162,246 in 2018; and 169,545 in 2019. Of the total sample, 43.7% of the children were BBFP. According to the Brazilian macroregions, 49.5% of the BBFP children and 61.6% of the NBFP children were from the Southeast region. There was a decrease in the number of children from the Midwest region for both BFP categories over the years (Table 1).

According to the complementary feeding indicators, a stationary trend in FI (APC 5.11%; *p* = 0.124), MMF (APC 2.53%; *p* = 0.309), and MDD (APC 2.60%; *p* = 0.455) was observed in the studied population over the period. In all of the years from 2015 to 2019, the prevalence of FI, MMF, and MDD were lower in the North and Northeast regions than in the South, Southeast, and Midwest regions. Increasing trends in FI were observed in the Northeast (APC 9.96%, *p* = 0.012) and Southeast regions (APC 6.11%, *p* = 0.018). Regarding MMF, increased prevalence was observed in the Northeast (APC 6.90%, *p* < 0.001), South (APC 2.09%, *p* = 0.026), and Midwest (APC 3.13%, *p* = 0.004) regions. Except for the South region (APC 2.75%, *p* = 0.005), all the other regions showed a stationary tendency for MDD. From 2015 to 2019, the prevalence of MMF (63.2% to 66.1%) and MDD (36.5% to 38.0%) in children aged 6–11 months were lower than those of older children (Table 2).

A stationary trend was observed for IFC (APC 0.54%, *p* = 0.550), VAFC (APC −0.53%, *p* = 0.749), and ZVF (APC −10.26%, *p* = 0.272) in the studied population. Except for the Midwest region, a decreasing prevalence of UFC was observed in the North, Northeast, Southeast, and South regions. For ZVF, there was a decrease in the Northeast (APC −14.54%, *p* = 0.004) and Southeast (APC −9.68%, *p* = 0.032) regions and an increase in the Midwest (APC 6.27%, *p* = 0.005) region. The North and Northeast regions showed a lower prevalence of IFC and VAFC and a higher prevalence of ZVF when compared to all other regions over the period. As for the age ranges, a lower prevalence of IFC, VAFC, and UFC was observed in children aged 6–11 months, while a higher prevalence of ZVF was found in children older than 12 months (Table 3).

There was a reduction in UNFC in the total sample (APC −7.44%, *p* = 0.006) and for all age ranges and macroregions, except in the South (APC −0.24, *p* = 0.375) and Midwest regions (APC 9.59, *p* = 0.002). A decreasing trend was observed related to ISSSF in the Southeast (APC −0.39, *p* = 0.021). Both EFF and EFF-without processed meat showed a decreasing trend in the North and an increase in Northeast, South, and Midwest regions (Table S3).

According to the complementary feeding indicators between BBFP and NBFP children, a decreasing trend in UFC (BBFP: APC −10.02%, *p* = 0.001 vs. NBFP: APC −9.34%, *p* = 0.002) and UNFC (BBFP: APC −8.58%, *p* = 0.006 vs. NBFP: APC −8.20%, *p* = 0.003) was observed. There was an increasing trend in ISSSF in BBFP children (APC 0.46%, *p* = 0.018). BBFP children displayed a lower prevalence of FI (17.2% to 20.8% vs. 23.5% to 25.2%), MMF (68.2% to 73.5% vs. 74.3% to 77.4%), MDD (38.5% to 42.6% vs. 47.0% to 48.5%), VAFC (58.2% to 60.0% vs. 67.6% to 66.6%), and EFF-without processed meat (78.1% to 80.5% vs. 80.9% to 81.2%) and a higher prevalence of UFC (61.7% to 54.9% vs. 55.6% to 46.4%), ZVF (11.2% to 8.8% vs. 7.2% to 6.3%), and UNFC (45.9% to 41.8% vs. 40.8% to 34.5%) than NBFP children in the studied period, which ranged from 2015 to 2019 (Table 4).

## 4. Discussion

The results revealed a stationary trend for most of the studied complementary feeding indicators, but with some variation according to the Brazilian macroregions. There was a decrease in the UFC over the years for both BBFP and NBFP children. There was lower prevalence of FI, MMF, MDD, and VAFC and a higher prevalence of ZVF in BBFP than in NBFP children. A lower prevalence of the same complementary feeding indicators was also observed in children under 1 year old and in those living in the North and Northeast regions.

The prevalence of complementary feeding indicators suggests the insufficient adherence to recommendations for adequate complementary feeding practices. A significant number of children were found to have been introduced to foods that are not recommended for their age range, with limited variety and consistency and inadequate sources of nutrients.

Almost 80% of the children showed an inadequate FI. It was higher than the latest global results where 31% did not reach the ISSSF at 6–8 months [18]. The prevalence of ISSSF estimated by the WHO protocol in this study was higher than 90% (results shown in the Supplementary Table S1). However, it is necessary to consider the differences between the Brazilian and WHO methods to assess infant feeding indicators. The higher prevalence of food introduction by the WHO protocol may be the result of considering the consumption of solid, semi-solid, or soft foods without differentiating between fresh/minimally processed and ultra-processed foods [21]. The Brazilian method assesses the intake of fruits and meals at the recommended frequency for the child’s age [19]. This national evaluation of FI is consistent with the Dietary Guidelines for Brazilian Children under 2 Years of Age, which recommends an increase in the number of meals according to age. Children aged six months must eat two snacks including fruits and one meal, and children aged from seven to 23 months must eat two snacks including fruits and two meals [16]. In the ENANI-2019, a qualitative component was added to the indicator construction, considering only the introduction of healthy foods, and the prevalence was 84.5% [24].

In the current study, MMF was higher according to age and the prevalence was similar to that observed in international studies. MMF assesses the age-appropriate frequency and consistency of food consumption as a proxy of adequate energy intake. Gatica-Domínguez et al., (2021) [25] evaluated national surveys conducted from 2010 to 2019 in 80 low and middle-income countries, and found 71.5% of MMF in Latin America and Caribbean countries, 72.5% in East Asia and the Pacific, 68.9% in Eastern Europe and Central Asia, and 62.9% in the Middle East and North Africa. However, the study used the WHO protocol, which takes into account a different number of meals according to age, differentiates between breastfed and non-breastfed children, and defines a minimum frequency of milk feeds [21].

The minimal diversity over the years has not changed, remaining below 50%, which shows a significant number of children who do not receive the diversity of nutrients needed for adequate growth and development [21]. This result is higher than the global average of 29% [18]. In the ENANI-2019, the MDD of children aged 6–23 months was 63.4%. They considered the WHO protocol with a cutoff point of five of eight food groups [5]. International studies performed in 2016 that assessed MDD (WHO protocol) found 19.5% in India [26], 12% in Ethiopia, and 38.5% in South Africa [27]. Proportions of MDD higher than 50% were found in Latin America and the Caribbean (56.5%), East Asia and the Pacific (50.6%), Eastern Europe, and Central Asia regions (50.3%) [25]. The Brazilian methodology for assessing MDD takes into account all six specified food groups and might result in a lower prevalence when compared to the other studies. Despite the methodological differences, these findings are not promising, given that the prevalence remained low.

The prevalence of IFC remained above 90.0% and was higher than that found in the ENANI-2019, which found a prevalence of 84.6% [24]. The ENANI-2019 considered the consumption of the following four food groups that are iron sources: flesh foods, eggs, legumes, and leafy vegetables, while the Brazilian Ministry of Health method analyzes the intake of one of the three defined food groups. These results show that nearly 15–20% of Brazilian children aged 6–23 months are not consuming iron-rich foods. Few international studies evaluated the consumption of iron-rich or iron-fortified foods by children aged 6–23 months according to the WHO protocol. Akalu et al., (2021) [28] analyzed a sample of 77,001 children aged 6–23 months from the Demographic and Health Surveys of Sub-Saharan Africa and found a prevalence of 42.1%. Studies conducted in Vietnam [29] and Malaysia [30] in 2014 found a prevalence of 89% and 92.3%, respectively. However, in the latest WHO publication, this WHO indicator, which includes iron-fortified or fortified foods at home, was excluded due to the difficulty of operationalizing it in household surveys [21]. Iron deficiency is still prevalent in Brazilian children. In the ENANI-2019, the prevalence of anemia in children aged 6–23 months was 19.9% [31]. Although the iron content in foods is affected by its bioavailability, it is strongly recommended that iron-source foods must be offered everyday during complementary feeding [16].

UFC and ZFV are the complementary feeding indicators that represent unhealthy eating practices. The decrease in the UFC indicator observed over the years raises questions about whether children may consume other ultra-processed foods not captured by the SISVAN questionnaire. In the ENANI-2019, the prevalence of ultra-processed food consumption was higher (80.5%) than what we found, and the second most consumed ultra-processed food was instant flours [5]. In any case, the prevalence of children consuming ultra-processed foods remained high. Even with national and international recommendations to avoid ultra-processed foods for children under two years of age, previous studies conducted between 2006 and 2016 also showed the early introduction of ultra-processed foods such as sandwich cookies, gelatin, sweets, savory snacks, and soft drinks in Brazilian children [3,4,6,32,33,34].

Another concern is that ultra-processed food is introduced prematurely in the first year of life and its consumption increases as children age. Souza et al., (2020) [32] and Cainelli et al., (2021) [33] also found higher proportions of children over one year old who consumed ultra-processed foods. This means that unhealthy eating habits start early and increase throughout life. Ultra-processed food consumption is related to a higher intake of fat, sugar, sodium, and additives [34], dental caries [35], high body fat [36], cardiovascular diseases [37], as well as reductions in breastfeeding duration and the consumption of healthy foods [38,39,40,41].

In the current study, children aged 6–11 months had a higher prevalence of ZVF than those above 12 months in all studied periods. However, these proportions were lower than those shown in the ENANI-2019, which observed a ZVF prevalence of 22.2% among children aged 6–23 months [24]. These findings are relevant to the discussion of public policies for greater access to fresh foods, while discouraging the consumption of ultra-processed foods, thus promoting the National Dietary Guidelines for Children [16] and the WHO Guideline recommendations on the complementary feeding of infants and young children 6–23 months of age [17].

The findings brought attention to inadequate complementary feeding as a public health problem in Brazil, as there was a stationary trend for all complementary feeding indicators of a healthy diet. The Brazilian political context during this period may have influenced these results. Following a period of implementing food and nutrition policies with the aim of achieving food and nutrition security, a severe recession began with a political crisis in 2016, which intensified in the following years. The reduction in budgets for social policies during this time period led to a significant rise in both misery and poverty [42].

In addition to the persistence of the prevalence of complementary feeding indicators, our findings revealed some vulnerability related to complementary feeding according to age range and Brazilian macroregions. Regarding age, the lowest prevalence of MMF, MDD, IFC, VAFC and the highest ZVF indicators were observed in children aged 6–11 months when compared to children older than one year. At 6 months of age, the introduction of food begins and progressively increases according to the child’s age. Moreover, this is a period full of expectations, doubts, and fears about what, how, and when to offer food to the child, which could influence these results [16,43].

Infants residing in the North and Northeast regions also had lower prevalence of FI, MMF, MDD, IFC, and VAFC and higher proportions of ZVF than the other regions. In the ENANI-2019, children living in the North region (54.8%) also had a lower prevalence of MDD compared to children from the other regions [5]. A previous study using data from the 2013 National Health Survey of 2451 children aged 12–23 months showed that children from the North and Northeast regions were more likely to not have a minimum dietary diversity [6]. The North and Northeast remain the regions with the lowest Human Development Index (HDI) rates in the country [44], which may explain these findings. These are the regions of the country that require further attention from policymakers. The results also draw attention to the Midwest region, which shows the opposite of the observed trends—an increase in UFC, ZVF, and UNFC and a decrease in FI over the years. A few studies have explored complementary feeding in children in the Midwest of Brazil. Spaniol et al., (2021) [34], in a sample of 538 children aged 6–24 months from primary health care units in the Federal District, found that one-third of the dietary energy intake came from processed and ultra-processed foods, most commonly instant flours, breakfast cereals and industrial baby foods, and sugar-sweetened milk beverages, contributing to higher intakes of saturated fat and lower intakes of dietary fiber. Conversely, the ENANI-2019 showed that the Midwest region had the highest prevalence of the combination of MDD with no consumption of ultra-processed foods (12.1%) when compared to the North (3.6%) and Northeast (6.0%) regions [5].

Our study pointed out the nutritional vulnerability of BBFP children. They had a lower prevalence of FI, MMF, MDD, and VACF and showed a prevalence of UFC 8.5% higher than NBFP children in 2019. In the study by Lignani et al., (2011) [45], BFP beneficiary families reported having more access to food diversity, but with higher amounts of ultra-processed foods. Cainelli et al., (2021) [33], using data collected in 2016 from 599 children aged six months to two years in a city in southeastern Brazil, found that children who received government benefits were more likely to consume ultra-processed foods than children who did not receive these benefits. In the UNICEF 2020 study of a representative sample of 1342 BFP beneficiary families, 72% of the children under two years of age had consumed some ultra-processed foods on the day before the survey. The main reasons given by these families were easier access, low cost, challenges in accessing healthy foods, and a lack of knowledge of what is healthy [46]. This highlights issues that need to be considered when developing public policies for this group and the general population.

It is relevant to discuss that families that are not BFP beneficiaries do not necessarily have better incomes. There is no available scientific literature reporting on BFP coverage at the national level. However, the Brazilian government, based on the estimated number of poor families from the national census in 2010, estimates that the BFP coverage was close to 100% between 2017 and 2021 [47].

BFP implementation has been shown to significantly reduce poverty, improve health care access, and decrease infant mortality rates [48]. Studies have shown that it provided access to food but did not improve diet quality. Contrary to the expectations, research suggests that it actually resulted in an increase in the consumption of highly processed foods [33,45]. Families benefiting from the program must follow the program’s conditionalities, which include antenatal care for pregnant women, immunization and nutritional monitoring for children under seven years of age, and a minimum school attendance for children and adolescents [49]. However, in addition to meeting these prerequisites, Damião et al., (2021) [50] point out the lack of individual and collective diagnoses in the daily routine of primary health units for the optimal nutritional monitoring of children and the limited comprehension by both health professionals and beneficiary families of the importance of nutritional surveillance in its full dimensions. All these findings emphasize the need for complementary policies to promote and provide access to healthy foods for the beneficiaries of the BFP.

This study is the first to use the SISVAN dataset to analyze the progress in complementary feeding practices between individuals who receive benefits from the BFP and those who do not. The analysis assessed the complementary feeding indicators based on Brazilian and WHO protocols, focusing on macroregions and age groups.

The generalizability of the results is limited by the fact that the sample is not representative of all Brazilian children and is affected by the SISVAN coverage. Ricci et al., (2023) [51] indicated that the total population coverage of the recording of food intake markers in SISVAN is still low, but they observed an upward trend with an annual increase rate of 45.6% per year from 2015 to 2019. Moreover, the geographic distribution of the children was similar to those in the general population, which showed that most Brazilians come from the Southeast region [52]. Similarly, in the ENANI-2019, most of the children were from the Southeast region and the percentage of BBFP children was slightly lower than in the present study (37.1% vs. 43.7%) [53].

Differences in the indicator national and international measurement methods limited the comparisons of our results with other countries. However, we used the method proposed by the Brazilian Ministry of Health, which is the current procedure for the surveillance of complementary feeding in Brazilian children, and we calculated the possible indicators according to WHO protocols. Also, we recommend further studies evaluating complementary feeding using the Brazilian Ministry of Health protocol to provide comparisons for surveillance in the country and to critically assess the use of the indicators in line with the current recommendations for appropriate complementary feeding.

In addition, it was not possible to evaluate other sociodemographic characteristics related to complementary feeding due to the use of a secondary dataset. We also recommend studies that explore other social and demographic factors, such as the income/socioeconomic status, maternal level of schooling, ethnicity/skin color, and employment status, among other aspects, to better understand the issues involved in providing adequate nutrition to children.

Another limitation is that a time series with fewer than seven points limits the power of the regression analysis. However, we were able to identify significant trends even with five points.

## 5. Conclusions

The study showed stationary trends related to the studied complementary feeding indicators, while the prevalence remained far from the recommendations in all of the analyzed periods. Although there are no targets for the complementary feeding indicators, all children are expected to have the right to adequate food and nutrition. In addition to identifying inadequate feeding practices at the beginning of the first year of life, the study highlighted the nutritional vulnerability of BBFP children. BBFP children had the lowest prevalence of indicators associated with a healthy diet and a higher prevalence of indicators associated with an unhealthy diet. This was also the case for children living in the North and Northeast regions. These findings emphasize the importance of nutritional surveillance in children and the need to improve public policies on food and nutrition for children under two years of age to promote equity, especially for beneficiaries of conditional cash transfer programs like the BFP.

## Figures and Tables

**Table 1:** Prevalence and 95% confidence intervals of Brazilian macroregions and age categories in children aged 6–23 months (*n* = 600,138), according to participation in the *Bolsa Família* Program. Data from the Brazilian Food and Nutritional Surveillance System (SISVAN), 2015–2019.

	Total (*n* = 600,138)	2015 (*n* = 39,197)	2016 (*n* = 101,636)	2017 (*n* = 127,514)	2018 (*n* = 162,246)	2019 (*n* = 169,545)	APC (95% CI)	p *
**Total sample**								
**BBFP**	43.7 (43.5; 43.8)	43.4 (42.9; 43.9)	42.7 (42.4; 43.0)	39.7 (39.4; 40.0)	44.0 (43.7; 44.2)	47.0 (46.8; 47.3)	4.45 (−9.22; 20.18)	0.396
**NBFP**	56.3 (56.2; 56.4)	56.6 (56.1; 57.1)	57.3 (57.0; 57.6)	60.3 (60.0; 60.5)	56.0 (55.8; 56.2)	53.0 (52.7; 53.2)	−3.49 (−13.19; 7.28)	0.363
**Brazilian macroregions**								
**BBFP**								
North	8.2 (8.1; 8.3)	15.5 (14.9; 16.0)	9.9 (9.6; 10.2)	6.1 (5.9; 6.3)	7.5 (7.3; 7.7)	7.7 (7.5; 7.8)	−32.01 (−64.71; 30.66)	0.156
Northeast	32.5 (32.3; 32.6)	35.9 (35.1; 36.6)	29.1 (28.7; 29.6)	21.6 (21.3; 22.0)	31.4 (31.1; 31.8)	41.4 (41.0; 41.7)	8.66 (−43.42; 108.69)	0.713
Southeast	49.5 (49.3; 49.7)	33.4 (32.7; 34.1)	50.3 (49.8; 50.8)	63.0 (62.6; 63.4)	50.6 (50.3; 51.0)	42.9 (42.6; 43.3)	12.64 (−38.63; 106.76)	0.577
South	6.4 (6.3; 6.5)	7.7 (7.3; 8.1)	5.7 (5.4; 5.9)	5.9 (5.7; 6.1)	7.5 (7.3; 7.7)	5.7 (5.5; 5.8)	−1.86 (−24.85; 28.16)	0.837
Midwest	3.4 (3.4; 3.5)	7.5 (7.2; 7.9)	4.9 (4.7; 5.2)	3.3 (3.1; 3.4)	2.9 (2.8; 3.0)	2.4 (2.2; 2.5)	−47.61 (−60.77; −30.03)	0.006
**NBFP**								
North	5.5 (5.4; 5.6)	10.9 (10.4; 11.3)	7.0 (6.9; 7.2)	4.1 (3.9; 4.2)	4.8 (4.6; 4.9)	5.1 (5.0; 5.3)	−35.38 (−67.06; 26.77)	0.131
Northeast	16.0 (15.8; 16.1)	15.0 (14.6; 15.5)	12.8 (12.5; 13.0)	10.0 (9.8; 10.2)	15.7 (15.4; 15.9)	23.6 (23.3; 23.9)	29.20 (−37.10; 165.39)	0.340
Southeast	61.6 (61.5; 61.8)	46.1 (45.4; 46.7)	62.5 (62.1; 62.9)	71.4 (71.1; 71.7)	61.8 (61.5; 62.1)	56.3 (56.0; 56.6)	9.51 (−27.19; 64.71)	0.530
South	12.1 (12.0; 12.2)	14.8 (14.4; 15.3)	10.2 (9.9; 10.4)	10.0 (9.8; 10.2)	13.9 (13.7; 14.1)	12.6 (12.3; 12.8)	1.96 (−33.47; 56.26)	0.894
Midwest	4.8 (4.8; 4.9)	13.1 (12.7; 13.6)	7.5 (7.3; 7.7)	4.5 (4.4; 4.7)	3.8 (3.7; 3.9)	2.3 (2.2; 2.4)	−61.09 (−69.30; −50.67)	0.001
**Age** (months)								
**BBFP**								
6–11	32.5 (32.3; 32.7)	33.3 (32.6; 34.0)	32.8 (32.4; 33.2)	31.4 (31.0; 31.8)	36.8 (36.4; 37.1)	29.1 (28.8; 29.4)	1.68 (−6.40; 10.47)	0.477
12–17	31.9 (31.7; 32.1)	32.0 (31.3; 32.7)	32.2 (31.7; 32.6)	30.8 (30.4; 31.2)	30.8 (30.4; 31.1)	33.4 (33.1; 33.7)	0.21 (−6.92; 7.88)	0.934
18–23	35.6 (35.4; 35.8)	34.7 (34.0; 35.5)	35.0 (34.5; 35.4)	37.8 (37.4; 38.2)	32.4 (32.1; 32.8)	37.5 (37.2; 37.8)	−0.80 (−7.22; 6.05)	0.726
**NBFP**								
6–11	39.8 (39.7; 40.0)	44.0 (43.3; 44.6)	42.8 (42.4; 43.2)	38.0 (37.6; 38.3)	38.4 (38.0; 38.7)	40.0 (39.7; 40.3)	−6.85 (−16.84; 4.35)	0.141
12–17	31.1 (31.0; 31.3)	31.4 (30.8; 32.0)	32.0 (31.7; 32.4)	32.0 (31.7; 32.4)	30.8 (30.5; 31.1)	30.0 (29.8; 30.3)	−2.92 (−7.64; 2.04)	0.155
18–23	29.0 (28.9; 29.2)	24.6 (24.0; 25.1)	25.1 (24.8; 25.5)	30.0 (29.7; 30.3)	30.8 (30.5; 31.1)	29.9 (29.7; 30.2)	15.93 (2.93; 30.57)	0.029

Notes: APC, annual prevalence change; BBFP, beneficiaries of the *Bolsa Família* Program; NBFP, non-beneficiaries of the *Bolsa Família* Program. * Prais–Winsten regression *p*-value.

**Table 2:** Prevalence and 95% confidence intervals of food introduction, minimum meal frequency and appropriate consistency and minimum dietary diversity indicators in children aged 6–23 months (*n* = 600,138), according to the Brazilian macroregions and age categories. Data from the Brazilian Food and Nutritional Surveillance System (SISVAN), 2015–2019.

	2015 (*n* = 39,197)		2016 (*n* = 101,636)		2017 (*n* = 127,514)		2018 (*n* = 162,246)		2019 (*n* = 169,545)		APC (95%CI)	p*
**Food Introduction (at 6–8 months) ^†^**												
**Total Sample**	21.5	(20.6; 22.4)	22.7	(22.1; 23.3)	24.1	(23.5; 24.7)	23.4	(22.9; 23.9)	23.6	(23.1; 24.1)	5.11 (−2.45; 13.25)	0.124
**Brazilian Macroregions**												
North	16.2	(13.9; 18.9)	15.3	(13.6; 17.2)	17.7	(15.3; 20.3)	16.1	(14.4; 17.9)	14.1	(12.6; 15.7)	−4.48 (−19.91; 14.40)	0.492
Northeast	14.0	(12.4; 15.7)	15.2	(14.1; 16.4)	15.1	(13.8; 16.5)	15.6	(14.7; 16.4)	17.6	(16.9; 18.4)	9.96 (3.91; 15.95)	0.012
Southeast	24.9	(23.4; 26.4)	25.7	(24.9; 26.5)	25.3	(24.6; 25.9)	26.8	(26.1; 27.4)	27.8	(27.1; 28.5)	6.11 (1.94; 10.45)	0.018
South	21.2	(18.9; 23.7)	23.1	(21.3; 25.1)	28.6	(26.7; 30.5)	26.9	(25.4; 28.3)	27.7	(26.1; 29.3)	17.24 (−1.30; 39.27)	0.061
Midwest	27.4	(24.8; 30.1)	26.4	(24.2; 28.7)	27.3	(24.8; 30.0)	23.2	(20.8; 25.8)	22.9	(19.9; 26.1)	−11.03 (−18.51; −2.86)	0.024
**Minimum Meal Frequency and Appropriate Consistency ^‡^**												
**Total Sample**	71.6	(71.1; 72.1)	75.6	(75.4; 75.9)	77.7	(77.4; 77.9)	75.6	(75.4; 75.8)	75.6	(75.3; 75.8)	2.53 (−3.94; 9.45)	0.309
**Brazilian Macroregions**												
North	63.2	(61.8; 64.5)	65.1	(64.0; 66.1)	65.4	(64.2; 66.6)	62.6	(61.6; 63.5)	64.1	(63.2; 65.0)	−0.72 (−4.54; 3.25)	0.600
Northeast	54.0	(53.0; 55.0)	56.2	(55.5; 57.0)	58.0	(57.3; 58.7)	58.6	(58.1; 59.1)	61.9	(61.4; 62.3)	6.90 (5.62; 8.19)	<0.001
Southeast	80.6	(80.0; 81.2)	82.4	(82.1; 82.7)	81.9	(81.6; 82.1)	82.0	(81.8; 82.3)	83.7	(83.4; 83.9)	1.23 (−0.02; 2.50)	0.052
South	81.0	(79.8; 82.1)	81.6	(80.7; 82.5)	84.1	(83.3; 84.8)	82.5	(81.9; 83.0)	84.6	(84.0; 85.1)	2.09 (0.46; 3.75)	0.026
Midwest	75.9	(74.6; 77.2)	78.5	(77.5; 79.5)	78.5	(77.3; 79.6)	79.5	(78.4; 80.6)	81.2	(80.0; 82.4)	3.13 (1.89; 4.38)	0.004
**Age** (months)												
6–11	63.2	(62.4; 63.9)	66.8	(66.4; 67.3)	69.1	(68.6; 69.5)	66.7	(66.3; 67.1)	66.1	(65.7; 66.5)	2.75 (−5.96; 12.27)	0.401
12–17	75.5	(74.8; 76.3)	79.8	(79.3; 80.2)	80.8	(80.4; 81.2)	79.1	(78.7; 79.4)	78.7	(78.3; 79.0)	1.68 (−4.21; 7.94)	0.440
18–23	78.7	(77.9; 79.5)	82.5	(82.0; 82.9)	83.7	(83.4; 84.1)	82.7	(82.4; 83.0)	82.3	(82.0; 82.6)	2.12 (−2.79; 7.29)	0.268
**Minimum Dietary Diversity**												
**Total Sample**	43.3	(42.8; 43.8)	45.9	(45.6; 46.2)	48.5	(48.2; 48.7)	45.9	(45.7; 46.1)	45.8	(45.5; 46.0)	2.60 (−6.75; 12.89)	0.455
**Brazilian Macroregions**												
North	28.9	(27.7; 30.2)	27.1	(26.2; 28.1)	25.1	(24.0; 26.2)	24.8	(24.0; 25.7)	25.7	(24.9; 26.5)	−7.14 (−16.04; 2.71)	0.101
Northeast	33.0	(32.1; 34.0)	33.6	(33.0; 34.3)	36.0	(35.3; 36.7)	35.2	(34.7; 35.7)	35.7	(35.3; 36.1)	4.86 (−0.57; 10.03)	0.051
Southeast	51.2	(50.4; 51.9)	52.3	(51.9; 52.7)	53.1	(52.8; 53.5)	51.8	(51.5; 52.2)	54.0	(53.6; 54.3)	1.53 (−0.43; 3.53)	0.090
South	46.0	(44.5; 47.4)	47.5	(46.4; 48.5)	46.9	(45.9; 47.8)	48.2	(47.5; 48.9)	48.7	(47.9; 49.5)	2.74 (1.56; 3.95)	0.005
Midwest	51.3	(49.7; 52.8)	48.4	(47.2; 49.6)	46.1	(44.8; 47.5)	47.4	(46.1; 48.7)	49.8	(48.3; 51.4)	−1.81 (−12.06; 9.63)	0.634
**Age** (months)												
6–11	36.5	(35.8; 37.3)	37.9	(37.4; 38.4)	40.7	(40.2; 41.2)	38.1	(37.7; 38.4)	38.0	(37.6; 38.3)	1.95 (−7.22; 12.04)	0.561
12–17	47.7	(46.8; 48.5)	51.0	(50.4; 51.5)	52.6	(52.1; 53.1)	50.4	(50.0; 50.8)	49.6	(49.2; 50.1)	1.56 (−7.49; 11.51)	0.633
18–23	47.8	(46.9; 48.7)	50.8	(50.3; 51.4)	52.8	(52.3; 53.3)	50.9	(50.5; 51.3)	50.2	(49.7; 50.6)	2.36 (−6.24; 11.76)	0.460

Notes: APC, annual prevalence change; CI, confidence interval. ^†^ Total sample *n* = 110,741 children (8075 in 2015; 19,806 in 2016; 22,467 in 2017; 30,993 in 2018; and 29,400 in 2019). ^‡^ Total sample *n* = 580,904 children (37,584 in 2015; 97,774 in 2016; 123,609 in 2017; 157,319 in 2018; and 164,628 in 2019). * Prais–Winsten regression *p*-value.

**Table 3:** Prevalence and 95% confidence intervals of iron-rich food, vitamin A-rich food, ultra-processed food, and zero vegetable or fruit consumption indicators in children aged 6–23 months (*n* = 600,138), according to the Brazilian macroregions and age categories. Data from the Brazilian Food and Nutritional Surveillance System (SISVAN), 2015–2019.

	2015 (*n* = 39,197)		2016 (*n* = 101,636)		2017 (*n* = 127,514)		2018 (*n* = 162,246)		2019 (*n* = 169,545)		APC (95%CI)	p *
**Iron-rich Food Consumption**												
**Total Sample**	90.0	(89.7; 90.3)	91.4	(91.2; 91.6)	92.6	(92.5; 92.7)	91.3	(91.2; 91.4)	91.1	(91.0; 91.2)	0.54 (−1.98; 3.12)	0.550
**Brazilian Macroregions**												
North	83.0	(81.9; 84.0)	81.3	(80.5; 82.2)	81.8	(80.9; 82.8)	81.1	(80.3; 81.9)	80.8	(80.0; 81.5)	−1.10 (−1.84; −0.16)	0.017
Northeast	84.2	(83.4; 84.9)	85.2	(84.7; 85.7)	86.9	(86.4; 87.3)	85.7	(85.4; 86.1)	86.7	(86.4; 87.0)	1.42 (−0.38; 3.24)	0.087
Southeast	94.4	(94.1; 94.8)	94.8	(94.6; 95.0)	94.6	(94.4; 94.7)	94.3	(94.1; 94.4)	94.8	(94.6; 94.9)	−0.06 (−0.43; 0.31)	0.635
South	92.8	(92.0; 93.5)	92.0	(91.4; 92.6)	92.8	(92.3; 93.3)	92.5	(92.1; 92.9)	92.9	(92.5; 93.3)	0.26 (−0.10; 0.63)	0.105
Midwest	91.7	(90.8; 92.5)	92.2	(91.6; 92.9)	92.5	(91.8; 93.2)	92.8	(92.1; 93.4)	93.5	(92.6; 94.2)	0.96 (0.73; 1.20)	0.003
**Age** (months)												
6–11	82.4	(81.8; 83.0)	84.6	(84.2; 85.0)	86.6	(86.3; 86.9)	84.3	(84.0; 84.6)	83.6	(83.3; 83.9)	0.59 (−4.13; 5.55)	0.721
12–17	94.3	(93.9; 94.7)	95.1	(94.9; 95.3)	95.2	(95.0; 95.4)	94.7	(94.6; 94.9)	94.3	(94.1; 94.5)	−0.10 (−1.28; 1.09)	0.810
18–23	95.7	(95.3; 96.1)	96.3	(96.1; 96.5)	96.5	(96.3; 96.7)	96.3	(96.1; 96.5)	95.9	(95.8; 96.1)	0.10 (−0.80; 1.01)	0.756
**Vitamin A-rich Food Consumption**												
**Total Sample**	63.5	(63.0; 64.0)	65.2	(64.9; 65.5)	66.2	(65.9; 66.5)	63.8	(63.5; 64.0)	63.5	(63.2; 63.7)	−0.53 (−5.20; 4.37)	0.749
**Brazilian Macroregions**												
North	50.4	(49.0; 51.8)	49.2	(48.2; 50.3)	46.5	(45.3; 47.8)	46.5	(45.5; 47.5)	48.8	(47.9; 49.8)	−2.81 (−10.21; 5.20)	0.336
Northeast	53.6	(52.5; 54.6)	53.8	(53.1; 54.5)	55.2	(54.5; 55.9)	55.5	(55.0; 56.0)	54.6	(54.1; 55.0)	1.79 (−0.72; 4.35)	0.109
Southeast	70.8	(70.1; 71.5)	70.8	(70.4; 71.2)	70.3	(70.0; 70.6)	68.5	(68.2; 68.8)	70.5	(70.2; 70.8)	−1.82 (−3.31; −0.31)	0.035
South	66.8	(65.4; 68.2)	67.5	(66.5; 68.5)	64.3	(63.4; 65.2)	65.6	(64.9; 66.2)	65.5	(64.8; 66.2)	−1.80 (−4.62; 1.09)	0.140
Midwest	70.5	(69.1; 71.9)	68.1	(67.0; 69.3)	64.2	(62.9; 65.5)	64.5	(63.3; 65.8)	66.4	(64.9; 67.9)	−3.90 (−10.88; 3.63)	0.192
**Age** (months)												
6–11	61.4	(60.6; 62.1)	62.8	(62.4; 63.3)	63.9	(63.5; 64.4)	61.3	(60.9; 61.6)	61.2	(60.8; 61.6)	−0.77 (−5.33; 4.01)	0.638
12–17	65.5	(64.6; 66.3)	67.5	(67.0; 68.0)	67.9	(67.5; 68.4)	65.7	(65.3; 66.2)	65.3	(64.9; 65.7)	−0.80 (−5.25; 3.86)	0.617
18–23	64.3	(63.4; 65.2)	65.8	(65.3; 66.4)	67.0	(66.5; 67.4)	64.9	(64.4; 65.3)	64.1	(63.7; 64.5)	−0.45 (−5.17; 4.50)	0.787
**Ultra-processed Food Consumption**												
**Total Sample**	58.2	(57.7; 58.7)	54.8	(54.4; 55.1)	52.5	(52.2; 52.8)	50.0	(49.8; 50.3)	50.4	(50.2; 50.6)	−8.66 (−11.88; −5.32)	0.004
**Brazilian Macroregions**												
North	60.7	(59.4; 62.1)	57.3	(56.3; 58.4)	56.1	(54.8; 57.3)	56.6	(55.6; 57.6)	52.6	(51.7; 53.6)	−5.63 (−8.43; −2.75)	0.009
Northeast	53.7	(52.7; 54.7)	53.8	(53.1; 54.5)	52.0	(51.3; 52.8)	48.4	(47.9; 48.9)	48.9	(48.5; 49.3)	−7.37 (−10.52; −4.11)	0.006
Southeast	61.1	(60.3; 61.8)	54.5	(54.1; 54.9)	51.6	(51.3; 51.9)	48.4	(48.1; 48.7)	49.7	(49.3; 50.0)	−11.67 (−18.61; −4.14)	0.017
South	59.3	(57.9; 60.7)	56.5	(55.4; 57.6)	57.1	(56.2; 58.0)	56.2	(55.5; 56.9)	55.7	(54.9; 56.5)	−2.59 (−4.47; −0.67)	0.023
Midwest	53.3	(51.8; 54.8)	54.4	(53.1; 55.6)	55.4	(54.1; 56.8)	57.0	(55.7; 58.3)	59.6	(58.1; 61.1)	6.39 (4.11; 8.73)	0.003
**Age** (months)												
6–11	42.2	(41.4; 43.0)	37.8	(37.3; 38.3)	35.7	(35.2; 36.1)	33.4	(33.1; 33.8)	32.7	(32.3; 33.0)	−13.55 (−17.86; −9.01)	0.003
12–17	64.7	(63.8; 65.5)	60.2	(59.6; 60.7)	57.1	(56.6; 57.6)	55.0	(54.5; 55.4)	54.6	(54.2; 55.0)	−9.43 (−13.90; −4.73)	0.008
18–23	72.9	(72.1; 73.7)	71.2	(70.6; 71.7)	66.0	(65.6; 66.5)	65.1	(64.7; 65.5)	64.9	(64.6; 65.3)	−7.35 (−11.58; −2.91)	0.014
**Zero Vegetable or Fruit Consumption**												
**Total Sample**	9.0	(8.7; 9.3)	8.0	(7.9; 8.2)	6.5	(6.4; 6.7)	7.2	(7.0; 7.3)	7.5	(7.4; 7.6)	−10.26 (−30.56; 15.99)	0.272
**Brazilian Macroregions**												
North	14.6	(14.6; 16.6)	15.9	(15.2; 16.7)	14.5	(13.6; 15.4)	15.4	(14.7; 16.1)	14.9	(14.3; 15.6)	−0.62 (−3.70; 2.56)	0.574
Northeast	13.5	(12.9; 14.2)	13.7	(13.3; 14.2)	11.8	(11.3; 12.3)	11.2	(10.9; 11.5)	11.1	(10.8; 11.3)	−14.54 (−19.51; −9.25)	0.004
Southeast	5.7	(5.3; 6.0)	5.2	(5.1; 5.4)	4.8	(4.7; 5.0)	4.9	(4.8; 5.0)	4.7	(4.5; 4.8)	−9.68 (−17.10; −1.60)	0.032
South	5.9	(5.3; 6.6)	6.7	(6.2; 7.3)	6.4	(6.0; 6.9)	6.0	(5.6; 6.3)	5.5	(5.1; 5.8)	−5.99 (−19.66; 10.00)	0.299
Midwest	6.5	(5.8; 7.3)	7.0	(6.4; 7.6)	7.0	(6.3; 7.7)	7.4	(6.7; 8.1)	7.2	(6.5; 8.1)	6.27 (3.62; 8.99)	0.005
**Age** (months)												
6–11	11.9	(11.3; 12.4)	10.7	(10.3; 11.0)	9.0	(8.8; 9.3)	10.0	(9.8; 10.3)	10.5	(10.3; 10.8)	−7.06 (−26.80; 18.01)	0.401
12–17	7.1	(6.6; 7.5)	6.4	(6.1; 6.6)	5.3	(5.0; 5.5)	5.6	(5.4; 5.8)	6.2	(6.0; 6.4)	−8.81 (−29.70; 18.29)	0.342
18–23	7.1	(6.7; 7.6)	6.4	(6.1; 6.7)	5.1	(4.9; 5.3)	5.2	(5.0; 5.4)	5.5	(5.3; 5.7)	−15.26 (−32.79; 6.84)	0.108

Notes: APC, annual prevalence change; CI, confidence interval. * Prais–Winsten regression *p*-value.

**Table 4:** Prevalence and 95% confidence intervals of complementary feeding indicators in children aged 6–23 months (*n* = 600,138), according to participation in the *Bolsa Família* Program. Data from the Brazilian Food and Nutritional Surveillance System (SISVAN), 2015–2019.

	2015 (*n* = 39,197)	2016 (*n* = 101,636)	2017 (*n* = 127,514)	2018 (*n* = 162,246)	2019 (*n* = 169,545)	APC (95% CI)	p *
**Brazilian Ministry of Health Indicators**							
**Food introduction (at 6–8 months)**							
**BBFP**	17.2 (16.4; 19.2)	18.7 (17.8; 19.6)	21.2 (20.3; 22.1)	20.6 (19.9; 21.3)	20.8 (20.0; 21.6)	11.60 (−1.43; 26.37)	0.067
**NBFP**	23.5 (22.4; 24.7)	24.9 (24.2; 25.6)	25.6 (24.9; 26.3)	25.5 (24.9; 26.1)	25.2 (24.5; 25.8)	3.85 (−2.47; 10.57)	0.151
**Minimum meal frequency and appropriate consistency**							
**BBFP**	68.2 (67.4; 68.9)	72.8 (72.4; 73.2)	75.4 (75.0; 75.8)	72.5 (72.2; 72.9)	73.5 (73.2; 73.9)	3.33 (−4.16; 11.40)	0.260
**NBFP**	74.3 (73.7; 74.9)	77.8 (77.4; 78.1)	79.2 (78.9; 79.5)	78.0 (77.7; 78.3)	77.4 (77.1; 77.6)	1.97 (−3.33; 7.56)	0.329
**Minimum dietary diversity**							
**BBFP**	38.5 (37.8; 39.2)	41.7 (41.2; 42.2)	44.8 (44.3; 45.2)	41.9 (41.5; 42.3)	42.6 (42.2; 42.9)	4.79 (−6.44; 17.38)	0.280
**NBFP**	47.0 (46.4; 47.7)	49.0 (48.6; 49.4)	50.9 (50.3; 51.2)	49.1 (48.7; 49.4)	48.5 (48.2; 48.9)	1.52 (−5.58; 9.16)	0.555
**Iron-rich food consumption**							
**BBFP**	88.9 (88.5; 89.4)	90.7 (90.5; 91.0)	92.0 (91.8; 92.2)	90.3 (90.0; 90.5)	91.0 (90.8; 91.2)	0.91 (−1.71; 3.60)	0.352
**NBFP**	90.8 (90.5; 91.2)	91.9 (91.7; 92.1)	93.0 (92.8; 93.2)	92.1 (91.9; 92.3)	91.2 (91.0; 91.4)	0.25 (−2.24; 2.81)	0.771
**Vitamin A-rich food consumption**							
**BBFP**	58.2 (57.5; 58.9)	60.5 (60.0; 60.9)	62.5 (62.1; 62.9)	59.8 (59.5; 60.2)	60.0 (59.6; 60.3)	1.09 (−5.11; 7.69)	0.625
**NBFP**	67.6 (67.0; 68.2)	68.8 (68.4; 69.1)	68.6 (68.3; 69.0)	66.9 (66.6; 67.2)	66.6 (66.3; 66.9)	−1.39 (−4.17; 1.46)	0.216
**Ultra-processed food consumption**							
**BBFP**	61.7 (61.0; 62.4)	59.4 (58.9; 59.9)	56.5 (56.1; 57.0)	53.0 (52.6; 53.4)	54.9 (54.6; 55.3)	−10.02 (−11.56; −8.46)	0.001
**NBFP**	55.6 (54.9; 56.2)	51.3 (50.9; 51.7)	49.8 (49.5; 50.2)	47.7 (47.4; 48.1)	46.4 (46.1; 46.7)	−9.34 (−11.93; −6.67)	0.002
**World Health Organization indicators**							
**Zero vegetable or fruit consumption**							
**BBFP**	11.2 (10.8; 11.7)	10.0 (9.7; 10.3)	8.2 (7.9; 8.4)	8.9 (8.7; 9.1)	8.8 (8.6; 9.0)	−12.87 (−28.98; 6.88)	0.121
**NBFP**	7.2 (6.8; 7.6)	6.6 (6.4; 6.8)	5.5 (5.3; 5.6)	5.8 (5.7; 6.0)	6.3 (6.2; 6.5)	−8.67 (−27.55; 15.12)	0.301
**Introduction of solid, semi-solid or soft food 6–8 months**							
**BBFP**	93.8 (92.8; 94.6)	93.5 (92.9; 94.0)	94.1 (93.6; 94.6)	94.2 (93.8; 94.6)	94.2 (93.7; 94.6)	0.46 (0.15; 0.76)	0.018
**NBFP**	94.5 (93.9; 95.1)	94.7 (94.3; 95.0)	95.0 (94.7; 95.4)	94.9 (94.6; 95.2)	94.6 (94.2; 94.9)	0.09 (−0.47; 0.67)	0.635
**Unhealthy food consumption**							
**BBFP**	45.9 (45.1; 46.6)	44.8 (44.4; 45.3)	42.7 (42.2; 43.1)	40.2 (39.8; 40.5)	41.8 (41.4; 42.1)	−8.58 (−11.33; −5.76)	0.006
**NBFP**	40.8 (40.2; 41.5)	37.6 (37.2; 38.0)	36.9 (36.6; 37.3)	35.7 (35.4; 36.0)	34.5 (34.2; 34.9)	−8.20 (−10.95; −5.36)	0.003
**Egg and/or flesh food consumption**							
**BBFP**	79.6 (79.0; 80.2)	80.4 (80.1; 80.8)	81.3 (80.9; 81.6)	79.9 (79.6; 80.2)	81.8 (81.5; 82.1)	0.73 (−0.46; 1.93)	0.146
**NBFP**	82.0 (81.5; 82.5)	82.3 (82.0; 82.6)	83.5 (83.2; 83.7)	83.0 (82.7; 83.2)	82.2 (81.9; 82.4)	0.31 (−1.61; 2.27)	0.644
**Egg and/or flesh food consumption (without processed meat)**							
**BBFP**	78.1 (77.5; 78.7)	79.1 (78.7; 79.4)	80.1 (79.7; 80.4)	78.8 (78.5; 79.1)	80.5 (80.2; 80.8)	1.02 (−0.39; 2.44)	0.106
**NBFP**	80.9 (80.4; 81.5)	81.4 (81.0; 81.7)	82.7 (82.4; 82.9)	82.2 (81.9; 82.4)	81.3 (81.1; 81.6)	0.45 (−1.80; 2.75)	0.574

Notes: APC, annual prevalence change; BBFP, beneficiaries of the *Bolsa Família* Program; CI, confidence interval; NBFP, non-beneficiaries of the *Bolsa Família* Program. * Prais-Winsten regression *p*-value.

## Data Availability

Data were obtained from Brazilian Ministry of Health and are available on request to Brazilian Ministry of Health.

## Supplementary Materials

The following supporting information can be downloaded at https://www.mdpi.com/article/10.3390/ijerph21070923/s1, Table S1: Definition of Brazilian Ministry of Health and WHO complementary feeding indicators; Table S2: Questions from the SISVAN’s form used to evaluate the consumption of food markers in children aged 6–23 months and questions used to construct the complementary feeding indicators; Table S3: Prevalence and 95% confidence intervals of introduction of solid, semi-solid or soft foods 6–8 months, unhealthy food consumption and egg and/or flesh food consumption indicators in children aged 6–23 months (*n* = 600,138), according to Brazilian macroregions and age categories. Data from the Brazilian Food and Nutritional Surveillance System (SISVAN), 2015–2019.

## References

[1] United Nations Children’s Fund (2019). The State of the World’s Children 2019. Children, Food and Nutrition: Growing Well in a Changing World.

[2] Lopes W.C., Marques F.K.S., Oliveira C.F.D., Rodrigues J.A., Silveira M.F., Caldeira A.P., Pinho L.D. (2018). Infant feeding in the first two years of life. Rev. Paul. Pediatr..

[3] Neves A.M., Madruga S.W. (2019). Complementary feeding, consumption of industrialized foods and nutritional status of children under 3 years old in Pelotas, Rio Grande do Sul, Brazil, 2016: A descriptive study. Epidemiol. Serv. Saúde.

[4] Bortolini G.A., Gubert M.B., Santos L.M.P. (2012). Food consumption Brazilian children by 6 to 59 months of age. Cad. Saúde Públ..

[5] Lacerda E.M.A., Bertoni N., Alves-Santos N.H., Carneiro L.B.V., Schincaglia R.M., Boccolini C.S., Castro I.R.R., Anjos L.A., Berti T.L., Kac G. (2023). Minimum dietary diversity and consumption of ultra-processed foods among Brazilian children 6–23 months of age. Cad. Saúde Pública.

[6] Rebouças A.G., Bernardino Í.d.M., Dutra E.R., Imparato J.C.P., Duarte D.A., Flório F.M. (2020). Factors associated with feeding practices among brazilian children aged 12 to 23 months old. Rev. Bras. Saúde Mater. Infant..

[7] Kambale R.M., Ngaboyeka G.A., Kasengi J.B., Niyitegeka S., Cinkenye B.R., Baruti A., Mutuga K.C., Van der Linden D. (2021). Minimum acceptable diet among children aged 6–23 months in South Kivu, Democratic Republic of Congo: A community-based cross-sectional study. BMC Pediatr..

[8] Bortolini G.A., Vitolo M.R., Gubert M.B., Santos L.M. (2015). Social inequalities influence the quality and diversity of diet in Brazilian children 6 to 36 months of age. Cad. Saúde Pública.

[9] Dallazen C., Silva S.A.D., Gonçalves V.S.S., Nilson E.A.F., Crispim S.P., Lang R.M.F., Moreira J.D., Tietzmann D.C., Vítolo M.R. (2018). Introdução de alimentos não recomendados no primeiro ano de vida e fatores associados em crianças de baixo nível socioeconômico. Cad. Saúde Pública.

[10] Bimpong K.A., Cheyuo E.K.E., Abdul-Mumin A., Ayanore M.A., Kubuga C.K., Mogre V. (2020). Mothers’ knowledge and attitudes regarding child feeding recommendations, complementary feeding practices and determinants of adequate diet. BMC Nutr..

[11] MEC Brasil (2004). Lei n. 10.836 de 09 de Janeiro de 2004. Cria o Programa Bolsa Família e dá Outras Providências.

[12] Ministério da Saúde (2022). Guia para a Organização da Vigilância Alimentar e Nutricional na Atenção Primária à Saúde.

[13] Mendes M., Marçal G., Rinaldi A., Bueno N., Florêncio T., Clemente A. (2022). Dietary patterns of children aged 6–24 months assisted by the Bolsa Família Program. Public Health Nutr..

[14] Saldiva S.R.D.M., Silva L.F.F., Saldiva P.H.N. (2010). Avaliação antropométrica e consumo alimentar em crianças menores de cinco anos residentes em um município da região do semiárido nordestino com cobertura parcial do programa bolsa família. Rev. Nutr..

[15] World Health Organization (2003). Global Strategy for Infant and Young Child Feeding.

[16] Ministério da Saúde (2019). Guia Alimentar para Crianças Brasileiras Menores de 2 Anos.

[17] World Health Organization (2023). Guideline for Complementary Feeding of Infants and Young Children 6–23 Months of Age.

[18] United Nations Children’s Fund (2020). Improving Young Children’s Diets During the Complementary Feeding Period.

[19] Ministério da Saúde (2015). Orientações para Avaliação de Marcadores de Consumo Alimentar na Atenção Básica.

[20] Mrejen M., Cruz M.V., Rosa L. (2023). O Sistema de Vigilância Alimentar e Nutricional (SISVAN) como ferramenta de monitoramento do estado nutricional de crianças e adolescentes no Brasil. Cad. Saúde Pública.

[21] World Health Organization, United Nations Children’s Fund (2021). Indicators for Assessing Infant and Young Child Feeding Practices: Definitions and Measurement Methods.

[22] StataCorp (2017). Stata Statistical Software: Release 15.

[23] Antunes J.L.F., Cardoso M.R.A. (2015). Uso da análise de séries temporais em estudos epidemiológicos. Epidemiol. Serv. Saúde.

[24] Federal University of Rio de Janeiro (2021). Prevalence of Feeding Indicators for Children under 5 Years of Age.

[25] Gatica-Domínguez G., Neves P.A., Barros A.J., Victora C.G. (2021). Complementary Feeding Practices in 80 Low-and Middle-Income Countries: Prevalence of and Socioeconomic Inequalities in Dietary Diversity, Meal Frequency, and Dietary Adequacy. J. Nutr..

[26] Beckerman-Hsu J.P., Kim R., Sharma S., Subramanian S.V. (2020). Dietary Variation among Children Meeting and Not Meeting Minimum Dietary Diversity: An Empirical Investigation of Food Group Consumption Patterns among 73,036 Children in India. J. Nutr..

[27] Heidkamp R.A., Kang Y., Chimanya K., Garg A., Matji J., Nyawo M., Craig H., Arimond M., Lyman A.L.T. (2020). Implications of Updating the Minimum Dietary Diversity for Children Indicator for Tracking Progress in the Eastern and Southern Africa Region. Curr. Dev. Nutr..

[28] Akalu Y., Yeshaw Y., Tesema G.A., Demissie G.D., Molla M.D., Muche A., Diress M., Tiruneh S.A. (2021). Iron-rich food consumption and associated factors among children aged 6–23 months in sub-Saharan Africa: A multilevel analysis of Demographic and Health Surveys. PLoS ONE.

[29] Tuan N.T., Withers M., Frongillo E.A., Hajeebhoy N. (2017). Estimates of the quality of complementary feeding among Vietnamese infants aged 6–23 months varied by how commercial baby cereals were classified in 24-h recalls. Matern. Child Nutr..

[30] Khor G.L., Tan S.Y., Tan K.L., Chan P.S., Amarra M.S. (2016). Compliance with WHO IYCF Indicators and Dietary Intake Adequacy in a Sample of Malaysian Infants Aged 6–23 Months. Nutrients.

[31] Castro I.R.R., Normando P., Farias D.R., Berti T.L., Schincaglia R.M., Andrade P., Bertoni N., Lacerda E.M.A., Anjos L.A., Boccolini C.S. (2023). Factors associated with anemia and vitamin A deficiency in Brazilian children under 5 years old: Brazilian National Survey on Child Nutrition (ENANI-2019). Cad. Saúde Pública.

[32] Souza J.P.D.O., Ferreira C.S., Lamounier D.M.B., Pereira L.A., Rinaldi A.E.M. (2020). Characterization of feeding of children under 24 months in units cared by the family health strategy. Rev. Paul. Pediatr..

[33] Cainelli E.C., Gondinho B.V.C., Palacio D.D.C., Oliveira D.B.D., Reis R.A., Cortellazzi K.L., Guerra L.M., Cavalcante D.F.B., Pereira A.C., Bulgareli J.V. (2021). Consumo de alimentos ultraprocessados por crianças e fatores socioeconômicos e demográficos associados. Einstein.

[34] Spaniol A.M., da Costa T.H.M., Souza A.M., Gubert M.B. (2021). Early consumption of ultra-processed foods among children under 2 years old in Brazil. Public Health Nutr..

[35] Zahid N., Khadka N., Ganguly M., Varimezova T., Turton B., Spero L., Sokal-Gutierrez K. (2020). Associations between Child Snack and Beverage Consumption, Severe Dental Caries, and Malnutrition in Nepal. Int. J. Environ. Res. Public Health.

[36] Costa C.S., Del-Ponte B., Assunção M.C.F., Santos I.S. (2018). Consumption of ultra-processed foods and body fat during childhood and adolescence: A systematic review. Public Health Nutr..

[37] Rauber F., Campagnolo P.D.B., Hoffman D.J., Vitolo M.R. (2015). Consumption of ultra-processed food products and its effects on children‘s lipid profiles: A longitudinal study. Nutr. Metab. Cardiovasc. Dis..

[38] Innes-Hughes C., Hardy L.L., Venugopal K., King L.A., Wolfenden L., Rangan A. (2011). Children‘s consumption of energy-dense nutrient-poor foods, fruit and vegetables: Are they related? An analysis of data from a cross sectional survey. Health Promot. J. Austr..

[39] Fonseca P.C.A., Ribeiro S.A.V., Andreoli C.S., de Carvalho C.A., Pessoa M.C., de Novaes J.F., Priore S.E., Franceschini S.D.C.C. (2019). Association of exclusive breastfeeding duration with consumption of ultra-processed foods, fruit and vegetables in Brazilian children. Eur. J. Nutr..

[40] Marçal G., Mendes M., Fragoso M., Florêncio T., Bueno N., Clemente A. (2021). Association between the consumption of ultra-processed foods and the practice of breast-feeding in children under 2 years of age who are beneficiaries of the conditional cash transfer programme, Bolsa Família. Public Health Nutr..

[41] Soares M.M., Juvanhol L.L., Ribeiro S.A.V., Franceschini S.D.C.C., Araújo R.M.A. (2021). Prevalence of processed and ultra-processed food intake in Brazilian children (6–24 months) is associated with maternal consumption and breastfeeding practices. Int. J. Food Sci. Nutr..

[42] Vasconcelos F.D.A.G.D., Machado M.L., Medeiros M.A.T.D., Neves J.A., Recine E., Pasquim E.M. (2019). Public policies of food and nutrition in Brazil: From Lula to Temer. Rev. Nutr..

[43] Ministério da Saúde (2009). Saúde da Criança: Nutrição Infantil: Aleitamento Materno e Alimentação Complementar.

[44] Programa das Nações Unidas para o Desenvolvimento, Instituto de Pesquisa Econômica Aplicada, Fundação João Pinheiro (2016). Desenvolvimento Humano nas Macrorregiões Brasileiras: 2016.

[45] Lignani J.B., Sichieri R., Burlandy L., Salles-Costa R. (2011). Changes in food consumption among the Programa Bolsa Família participant families in Brazil. Public Health Nutr..

[46] United Nations Children’s Fund (2021). Alimentação na Primeira Infância: Conhecimentos, Atitudes e Práticas de Beneficiários do Programa Bolsa Família.

[47] Secretaria de Avaliação, Gestão da Informação e Cadastro Único (SAGICAD) Percentual de Cobertura das Famílias do Bolsa Família com Base na Estimativa de Famílias Pobres do Censo IBGE 2010. https://aplicacoes.cidadania.gov.br.

[48] Neves J.A., Vasconcelos F.D.A.G.D., Machado M.L., Recine E., Garcia G.S., Medeiros M.A.T.D. (2022). The Brazilian cash transfer program (Bolsa Família): A tool for reducing inequalities and achieving social rights in Brazil. Glob. Public Health.

[49] Diário Oficial da União (2023). Medida Provisória n. 1.164 de 02 de Março de 2023. Institui o Programa Bolsa Família e Altera a Lei nº 8.742, de 7 de Dezembro de 1993, que Dispõe Sobre a Organização da Assistência Social, e a Lei nº 10.820, de 17 de Dezembro de 2003, que Dispõe Sobre a Autorização para Desconto em Folha de Pagamento.

[50] Damião J.J., Lobato E., Silva J.P., Silva C.V.C., Castro L.M.C., Maldonado L.A., Ribeiro A.A. (2021). Condicionalidades de saúde no Programa Bolsa Família e a vigilância alimentar e nutricional: Narrativas de profissionais da atenção primária à saúde. Cad. Saúde Pública.

[51] Ricci J.M.S., Romito A.L.Z., Silva S.A.D., Carioca A.A.F., Lourenço B.H. (2023). Marcadores do consumo alimentar do Sisvan: Tendência temporal da cobertura e integração com o e-SUS APS, 2015–2019. Ciênc. Saúde Coletiva.

[52] Instituto Brasileiro de Geografia e Estatística (2020). Pesquisa Nacional por Amostra de Domicílios Contínua 2012/2019: Características Gerais dos Domicílios e dos Moradores 2019.

[53] Federal University of Rio de Janeiro (2021). Sociodemographic Characteristics: Demographic, Socioeconomic and Food Security Aspects.

