# Supplementary material for: Trends in Complementary Feeding Indicators in Children Aged 6–23 Months According to Participation in a Conditional Cash Transfer Program: Data from the Brazilian Food and Nutrition Surveillance System, 2015–2019
Source: Int J Environ Res Public Health. 2024 Jul 15;21(7):923. doi: 10.3390/ijerph21070923 (PMC11276628; doi:10.3390/ijerph21070923)
Supplement: Supplementary file 1 [file ijerph-21-00923-s001.zip › ijerph-3003372-supplementary.pdf]

International Journal of *Environmental Research and Public Health*

MDPI

Supplementary Material

Table S1. Definition of Brazilian Ministry of Health and WHO complementary feeding indicators.

Indicator	Definition
**Brazilian Ministry of Health complementary feeding indicators †**	
**Indicator**	**Definition**
Food introduction (at 6–8 months) (FI)	Percentage of infants aged six months who consumed fruits twice and one meal during the previous day.<br>Percentage of infants aged 7–8 months who consumed fruits twice and two meals during the previous day.
Minimum meal frequency and appropriate consistency (MMF)	Percentage of infants aged 6 months who consumed at least one meal in the appropriate consistency (mashed or in pieces) during the previous day;<br>Percentage of children aged 7–23 months who received at least two meals in the appropriate consistency (mashed or in pieces) during the previous day.
Minimum dietary diversity (MDD)	Percentage of children aged 6–23 months who consumed the following six food groups during the previous day:<br>(i) breast milk or dairy products;<br>(ii) fruits, vegetables, and greens;<br>(iii) orange-colored vegetables/fruits or dark green leaves;<br>(iv) meat/eggs;<br>(v) legumes; and<br>(vi) cereals/tubers (rice, potatoes, yams, cassava, flour or noodles-not instant).
Iron-rich foods consumption (IFC)	Percentage of children aged 6–23 months who consumed at least one of the following three food groups during the previous day:<br>(i) meat/eggs;<br>(ii) legumes; and<br>(iii) liver.
Vitamin A-rich foods consumption (VAFC)	Percentage of children aged 6–23 months who consumed orange-colored vegetables/fruits or dark green leaves during the previous day.
Ultra-processed food consumption (UFC)	Percentage of children aged 6–23 months who consumed at least one of the following four food groups during the previous day:<br>(i) hamburgers/cold meats (ham, bologna, salami, hot dog);<br>(ii) sugar-sweetened beverages (soda, juice, powdered fruit juice, coconut water, guarana syrup, fruit juice with added sugar);<br>(iii) instant noodles, packaged snacks, or crackers; and<br>(iv) sandwich cookies, sweets or candies (candies, lollipops, chewing gum, caramel, jelly).
**WHO complementary feeding indicators ‡**	
**Indicator**	**Definition**
Introduction of solid, semi-solid, or soft foods 6–8 months (ISSSF)	Percentage of infants aged 6–8 months who consumed solid, semi-solid, or soft foods during the previous day.
Unhealthy food consumption (UNFC)	Percentage of children aged 6–23 months who consumed selected sentinel unhealthy foods (sweet foods and fried and salty foods) during the previous day.
Egg and/or flesh food consumption (EFF)	Percentage of children aged 6–23 months who consumed egg and/or flesh food during the previous day. This indicator was also calculated without considering consumption of processed meat.
Zero vegetable or fruit consumption (ZVF)	Percentage of children aged 6–23 months who did not consume any vegetables or fruits during the previous day.

Source: † Ministério da Saúde, 2015 [19]. ‡ WHO and UNICEF, 2021 [21].

Table S2. Questions from the SISVAN's form used to evaluate the consumption of food markers in children aged 6–23 months and questions used to construct the complementary feeding indicators.

	**Questions from the SISVAN's form for children aged 6–23 months**
1	Was the child breastfed yesterday? *(Yes/No/Don't know)*
2A	Did the child eat whole, sliced or mashed fruit yesterday? *(Yes/No/Don't know)*
2B	If yes. How many times? *(Once/Twice/Three times or more/Don't know)*
3A	Did the child eat meals yesterday? *(Yes/No/Don't know)*
3B	If yes. How many times? *(Once/Twice/Three times or more/Don't know)*
3C	If yes. How was it offered? (*In pieces/Mashed/Sifted/Blended/Liquid/Don't know*)
4	Did the child eat porridge with cow's milk yesterday? *(Yes/No/Don't know)*
5	Did the child eat yogurt yesterday? *(Yes/No/Don't know)*
6	Did the child eat vegetables (do not consider those used to make sauce, or potatoes, cassava, yam and other roots) yesterday? *(Yes/No/Don't know)*
7	Did the child eat orange-colored vegetables/fruits or dark green leaves yesterday? *(Yes/No/Don't know)*
8	Did the child eat leafy vegetables yesterday? *(Yes/No/Don't know)*
9	Did the child eat meat (beef, pork, chicken, fish, organ meats, others) or eggs yesterday? *(Yes/No/Don't know)*
10	Did the child eat liver yesterday? *(Yes/No/Don't know)*
11	Did the child eat beans yesterday? *(Yes/No/Don't know)*
12	Did the child eat rice, potatoes, yams, cassava, flour or noodles (not instant) yesterday? *(Yes/No/Don't know)*
13	Did the child eat hamburgers/cold meats (ham, bologna, salami, hot dogs) yesterday? *(Yes/No/Don't know)*
14	Did the child drink sugar-sweetened beverages (soda, juice, powdered fruit juice, coconut water, guarana syrup, fruit juice with added sugar) yesterday? *(Yes/No/Don't know)*
15	Did the child eat instant noodles, packaged snacks, or crackers yesterday? *(Yes/No/Don't know)*
16	Did the child eat sandwich cookies, sweets or candies (candies, lollipops, chewing gum, caramel, jelly) yesterday? *(Yes/No/Don't know)*

**Questions in SISVAN's questionnaire used in each indicator construction**	
Food introduction at 6–8 months (FI)	2A; 2B; 3A; 3B
Minimum meal frequency and appropriate consistency (MMF)	3A; 3B; 3C
Minimum dietary diversity (MDD)	1; 4; 6; 7; 8; 9; 11; 12
Iron-rich food consumption (IFC)	9; 10; 11
Vitamin A-rich food consumption (VAFC)	7
Ultra-processed food consumption (UFC)	13; 14; 15; 16
Zero vegetable or fruit consumption (ZVF)	2A; 6; 7; 8
Introduction of solid, semi-solid, or soft foods 6–8 months (ISSSF)	2A; 3A; 4; 5; 6; 7; 8; 9; 10; 11; 12; 13; 15; 16
Unhealthy food consumption (UNFC)	15; 16
Egg and/or flesh food consumption (EFF)	9; 10; 13
Egg and/or flesh food consumption (EFF)- without processed meat	9; 10

Source: Ministério da Saúde, 2015 [19].

Table S3. Prevalence and 95% confidence intervals of introduction of solid, semi-solid or soft foods 6–8 months, unhealthy food consumption and egg and/or flesh food consumption indicators in children aged 6–23 months (*n* = 600,138), according to Brazilian macroregions and age categories. Data from the Brazilian Food and Nutritional Surveillance System (SISVAN), 2015–2019.

	2015 (*n* = 39,197)		2016 (*n* = 101,636)		2017 (*n* = 127,514)		2018 (*n* = 162,246)		2019 (*n* = 169,545)		APC (95%CI)	*p* *
**Introduction of solid, semi-solid or soft foods 6–8 months †**												
**Total sample**	94.3	(93.7; 94.7)	94.3	(93.9; 94.6)	94.7	(94.4; 95.0)	94.6	(94.4; 94.9)	94.4	(94.2; 94.7)	0.14 (−0.28; 0.56)	0.377
**Brazilian macroregions**												
North	89.1	(86.8; 91.0)	87.9	(86.2; 89.5)	89.8	(87.7; 91.6)	89.3	(87.8; 90.7)	88.3	(86.8; 89.7)	0.26 (−1.32; 1.87)	0.639
Northeast	93.7	(92.5; 94.8)	94.2	(93.5; 94.9)	93.9	(93.0; 94.7)	94.5	(93.9; 95.0)	93.7	(93.2; 94.2)	0.21 (−0.04; 0.46)	0.070
Southeast	95.7	(95.0; 96.3)	95.4	(95.0; 95.8)	95.3	(95.0; 95.6)	95.0	(94.7; 95.4)	95.4	(95.0; 95.7)	−0.39 (−0.64; −0.14)	0.021
South	95.8	(94.5; 96.8)	94.8	(93.8; 95.7)	94.7	(93.7; 95.5)	95.5	(94.8; 96.1)	95.6	(94.8; 96.2)	0.09 (−1.23; 1.44)	0.839
Midwest	93.1	(91.4; 94.4)	91.5	(89.9; 92.8)	92.9	(91.3; 94.3)	93.9	(92.3; 95.1)	95.6	(93.9; 96.9)	1.95 (−0.27; 4.21)	0.068
**Unhealthy food consumption**												
**Total sample**	43.0	(42.5; 43.5)	40.7	(40.4; 41.0)	39.2	(39.0; 39.5)	37.7	(37.4; 37.9)	37.9	(37.7; 38.2)	−7.44 (−10.71; −4.05)	0.006
**Brazilian macroregions**												
North	44.5	(43.1; 45.8)	41.2	(40.2; 42.3)	40.6	(39.4; 41.8)	42.1	(41.1; 43.0)	39.6	(38.7; 40.5)	−3.92 (−9.41; 1.90)	0.119
Northeast	40.3	(39.3; 41.3)	40.0	(39.3; 40.7)	39.3	(38.6; 40.0)	35.9	(35.4; 36.4)	36.2	(35.8; 36.6)	−7.88 (−11.56; −4.05)	0.008
Southeast	44.3	(43.6; 45.1)	40.1	(39.7; 40.5)	38.1	(37.8; 38.4)	36.1	(35.8; 36.4)	37.1	(36.7; 37.4)	−10.19 (−16.89; −2.94)	0.022
South	45.4	(44.0; 46.9)	44.9	(43.8; 46.0)	45.5	(44.5; 46.4)	45.0	(44.3; 45.7)	45.0	(44.2; 45.8)	−0.24 (−0.96; 0.49)	0.375
Midwest	39.7	(38.2; 41.2)	41.6	(40.4; 42.8)	43.0	(41.7; 44.4)	44.5	(43.2; 45.9)	47.5	(46.0; 49.1)	9.59 (7.80; 11.40)	0.002
**Age** (months)												
6–11	27.7	(27.0; 28.4)	25.0	(24.5; 25.4)	23.9	(23.5; 24.3)	22.9	(22.6; 23.2)	22.2	(21.8; 22.5)	−11.37 (−15.69; −6.83)	0.005
12–17	49.0	(48.1; 49.9)	45.6	(45.0; 46.1)	43.2	(42.7; 43.6)	41.8	(41.4; 42.2)	41.1	(40.7; 41.5)	−9.61 (−13.79; −5.22)	0.007
18–23	57.2	(56.3; 58.1)	56.0	(55.5; 56.6)	51.9	(51.4; 52.3)	51.3	(50.9; 51.7)	51.4	(51.0; 51.8)	−6.92 (−11.45; −2.16)	0.020
**Egg and/or flesh food consumption**												
**Total sample**	81.0	(80.6; 81.3)	81.5	(81.3; 81.7)	82.6	(82.3; 82.8)	81.6	(81.5; 81.8)	82.0	(81.8; 82.2)	0.56 (−0.83; 1.97)	0.294
**Brazilian macroregions**												
North	75.2	(74.0; 76.4)	74.3	(73.3; 75.2)	73.9	(72.8; 75.0)	73.8	(72.9; 74.7)	73.5	(72.6; 74.3)	−1.19 (−1.96; −0.41)	0.017
Northeast	75.3	(74.3; 76.2)	76.6	(76.0; 77.2)	77.7	(77.1; 78.3)	76.7	(76.3; 77.1)	78.3	(78.0; 78.7)	1.61 (0.04; 3.21)	0.047
Southeast	84.8	(84.2; 85.4)	83.9	(83.6; 84.2)	84.0	(83.8; 84.2)	83.7	(83.5; 84.0)	84.8	(84.6; 85.1)	−0.09 (−1.22; 1.06)	0.818
South	83.1	(82.0; 84.1)	82.0	(81.1; 82.8)	84.0	(83.3; 84.7)	83.8	(83.3; 84.4)	84.3	(83.7; 84.8)	1.43 (0.51; 2.35)	0.015
Midwest	83.6	(82.5; 84.7)	83.3	(82.4; 84.2)	84.0	(83.0; 85.0)	85.8	(84.8; 86.7)	85.7	(84.5; 86.7)	2.03 (0.73; 3.35)	0.015
**Age** (months)												
6–11	69.4	(68.7; 70.1)	69.8	(69.3; 70.2)	70.9	(70.4; 71.3)	70.0	(69.6; 70.3)	69.8	(69.4; 70.1)	0.33 (−1.61; 2.31)	0.628
12–17	86.9	(86.3; 87.5)	87.3	(87.0; 87.7)	87.6	(87.3; 87.9)	87.1	(86.8; 87.4)	86.8	(86.6; 87.1)	−0.10 (−1.09; 0.89)	0.763
18–23	90.2	(89.6; 90.7)	90.6	(90.2; 90.9)	90.3	(90.0; 90.6)	90.3	(90.0; 90.5)	90.2	(89.9; 90.4)	−0.03 (−0.45; −0.39)	0.831
**Egg and/or flesh food consumption (without processed meat)**												
**Total sample**	79.7	(79.3; 80.1)	80.4	(80.1; 80.6)	81.6	(81.4; 81.9)	80.7	(80.5; 80.9)	80.9	(80.8; 81.1)	0.76 (−0.94; 2.49)	0.251
**Brazilian macroregions**												
North	73.4	(72.2; 74.6)	72.7	(71.8; 73.7)	72.3	(71.1; 73.4)	72.0	(71.0; 72.8)	72.1	(71.2; 72.9)	−1.50 (−1.75; −1.25)	<0.001
Northeast	73.9	(73.0; 74.8)	75.2	(74.6; 75.8)	76.5	(75.9; 77.1)	75.5	(75.1; 76.0)	77.3	(76.9; 77.6)	1.93 (0.34; 3.53)	0.030
Southeast	83.8	(83.2; 84.3)	83.0	(82.7; 83.3)	83.1	(82.9; 83.4)	82.9	(82.6; 83.1)	83.8	(83.5; 84.0)	−0.04 (−1.07; 1.00)	0.902
South	81.7	(80.5; 82.8)	80.9	(80.1; 81.8)	83.2	(82.4; 83.9)	83.1	(82.5; 83.6)	83.4	(82.9; 84.0)	1.87 (0.69; 3.05)	0.015
Midwest	82.8	(81.6; 83.9)	82.4	(81.5; 83.3)	83.2	(82.1; 84.2)	85.1	(84.1; 86.0)	84.5	(83.3; 85.6)	2.03 (0.71; 3.37)	0.016
**Age** (months)												
6–11	68.4	(67.6; 69.1)	68.8	(68.4; 69.3)	70.1	(69.7; 70.5)	69.2	(68.8; 69.6)	69.0	(68.6; 69.3)	0.54 (−1.57; 2.70)	0.478
12–17	85.4	(84.6; 86.0)	86.2	(85.8; 86.5)	86.7	(86.3; 87.0)	86.1	(85.8; 86.4)	85.8	(85.5; 86.1)	0.19 (−1.29; 1.70)	0.707
18–23	88.9	(88.3; 89.5)	89.2	(88.8; 89.5)	89.2	(88.9; 89.5)	89.1	(88.8; 89.4)	88.8	(88.6; 89.1)	−0.08 (−0.58; 0.42)	0.650

Notes: APC, annual prevalence change; CI, confidence interval. † Total sample *n* = 111,895 children (8181 in 2015; 20,143 in 2016; 22,696 in 2017; 31,229 in 2018; and 29,646 in 2019). * Prais-Winsten regression *p*-value.
